# A Trial of the Efficacy and Cost of Water Delivery Systems in San Francisco Bay Area Middle Schools, 2013

**DOI:** 10.5888/pcd13.160108

**Published:** 2016-07-07

**Authors:** Anisha I. Patel, Anna H. Grummon, Karla E. Hampton, Ariana Oliva, Charles E. McCulloch, Claire D. Brindis

**Affiliations:** Author Affiliations: Anna H. Grummon, Division of General Pediatrics, University of California, San Francisco, California; Karla E. Hampton, Enigami Ventures, LLC, Richmond, California; Ariana Oliva, California Food Policy Advocates, Oakland, California; Charles E. McCulloch, Division of Biostatistics, University of California, San Francisco, California; Claire D. Brindis, Division of General Pediatrics, Philip R. Lee Institute for Health Policy Studies, and Adolescent and Young Adult Health National Resource Center, University of California, San Francisco, California.

## Abstract

**Introduction:**

US legislation requires that schools offer free drinking water where meals are served. However, little information is available about what types of water delivery systems schools should install to meet such requirements. The study objective was to examine the efficacy and cost of 2 water delivery systems (water dispensers and bottleless water coolers) in increasing students’ lunchtime intake of water in low-income middle schools.

**Methods:**

In 2013, twelve middle schools in the San Francisco Bay Area participated in a cluster randomized controlled trial in which they received 6 weeks of promotional activities, received provision of cups, and were assigned to 1 of 2 cafeteria water delivery systems: water dispensers or bottleless water coolers (or schools served as a control). Student surveys (n = 595) and observations examined the interventions’ effect on students’ beverage intake and staff surveys and public data assessed intervention cost.

**Results:**

Analysis occurred from 2013 through 2015. Mixed-effects logistic regression, accounting for clustering and adjustment for student sociodemographic characteristics, demonstrated a significant increase in the odds of students drinking water in schools with promotion plus water dispensers and cups (adjusted odds ratio = 3.1; 95% confidence interval, 1.4–6.7; *P* = .004) compared with schools with traditional drinking fountains and no cups or promotion. The cost of dispenser and bottleless water cooler programs was similar ($0.04 per student per day).

**Conclusion:**

Instead of relying on traditional drinking fountains, schools should consider installing water sources, such as plastic dispensers with cups, as a low-cost, effective means for increasing students’ water intake.

## Introduction

Obesity prevention in childhood can prevent adult obesity and related illnesses (1). Intake of sugar-sweetened beverages (SSBs), drinks with added sugar, is a major contributor to obesity, obesity-related comorbidities (2), and dental caries (3).

Health promotion efforts have focused on reducing SSB access in schools, where children spend substantial time (4), but only recently have interventions emphasized improving access to drinking water (5–7). The Institute of Medicine and the Centers for Disease Control and Prevention recommend that schools improve the availability of potable and free drinking water as a low-cost obesity prevention strategy (8,9). Because children are not properly hydrated, which impairs their learning capability, improvements in school water access may also improve students’ readiness to learn (10).

The 2010 Healthy, Hunger-Free Kids Act required that schools participating in federal meal programs provide free potable water in places where meals are served (11). Most schools meet these requirements through drinking fountains (12), which students may avoid because of concerns about their cleanliness and sub-standard water quality (13,14). Studies suggest that students drink more water when schools supply cups and provide water sources that are more appealing than traditional fountains (5–7,15). However, schools have little information about the efficacy and cost of providing more appealing water sources.

This study’s objective was to conduct a cluster, randomized controlled trial in 12 low-income middle schools to examine how offering and promoting 1) water dispensers with cups or 2) a bottleless water cooler with cups in school cafeterias influences students’ lunchtime intake of water and other beverages compared with drinking fountains without cups or promotion. The hypothesis was that a bottleless water cooler, cups, and promotion would be most effective at increasing students’ water intake.

## Methods

### Participants

Eligible schools were middle or junior high schools (serving students in grades 6 through 8 or grades 7 and 8; excluding special educational, vocational, and alternative schools) in the San Francisco Bay Area of California. Low-income and minority children have poorer beverage intake habits (16) and higher rates of obesity than high-income and nonminority children (17); therefore, only schools serving 50% or more of students eligible for free and reduced-price meals (FRPM) through the National School Lunch Program and 50% or more of students of Latino or African American race/ethnicity were eligible. The study team approached districts with schools meeting eligibility criteria after receiving approval from districts and the Committee for Human Subjects at the University of California, San Francisco. Schools were ineligible if they had or were scheduled to receive hydration stations (drinking water sources in which students can fill reusable water bottles) or tap water dispensers from a source outside of the study. Across study arms, schools were matched on the basis of school district, student enrollment, percentage of Latino students, percentage of African American students, and percentage of FRPM-eligible students. Three counties included districts large enough to recruit matched schools from within a single district. In a fourth county with smaller school districts, matched schools were recruited from 2 adjacent districts. If a school declined participation, researchers sequentially approached remaining schools from the list of matched schools in the county. Once 3 schools within each district/county agreed to participate, a random number generator was used to allocate schools to receive 1 of 3 conditions: 1) dispenser intervention, 2) cooler intervention, or 3) control. This approach was used so that intervention and control schools could be comparable in terms of geographic location, school district, and weather.

### Intervention

Intervention components drew from previous research (6), the Transtheoretical Model, the 4 Ps of social marketing, and Brennan et al’s conceptual framework of policy and environmental strategies to prevent childhood obesity (18). In spring 2013, intervention schools received water testing and remediation for lead on the basis of Environmental Protection Agency (EPA) recommendations (19). Of the 12 study schools, 2 had first draw samples (after overnight stagnation but before flushing) with lead levels that were detectable but below the EPA action limit. In the school in which 30 seconds of flushing led to undetectable lead, cafeteria staff flushed water before filling dispensers. In another school in which flushing slightly decreased lead (16 ppb to 13 ppb), a filter was installed to remove lead. Intervention schools also received cups for lunchtime use and either a Service Ideas NSF-certified dispenser (https://www.serviceideas.com/Main/product.aspx?product=CBDT3SS&um=Each&bh=03000201) or a Culligan bottleless water cooler (http://culligantulsa.com/bottled-water/#bottle-free-water-cooler) to provide chilled water in the cafeteria. Researchers also implemented weekly promotion for 6 weeks (ie, posting of signage, schoolwide audio announcements, parent newsletter messages, prizes for students observed drinking water) focused on a theme related to the benefits of drinking water (ie, water is safe, water is healthy, water is tasty, water is cheap, water is easy, and water is green). Control schools did not receive any intervention and had only traditional water fountains. Midway through the study period, 1 intervention and 1 control school received a reusable water bottle filling station independent of this study in a common hallway and cafeteria, respectively.

### Data collection

Within each school, researchers used a random number generator to select 60 students, stratified by grade level, to complete surveys about their lunchtime beverage intake. Surveys, developed on the basis of a previously validated survey (20) and the research team’s previous studies, were administered at preintervention (February and March 2013) and postintervention (May and June 2013) to estimate the intervention’s effect on students’ intake of beverages. Researchers determined the sample size on the basis of a design effect of 2.1, an intraclass correlation of 0.05 on the basis of previous studies, and 80% power at the 5% significance level to detect moderate changes in the percentage of students who drank free water at lunchtime in cafeterias (6% in control schools to 20% in intervention schools).

Researchers also conducted schoolwide observations during lunchtime 1 week before the promotion, during each week of the 6-week promotional period, and 1 week after the promotion (21) to document the proportion of students accessing water sources in cafeterias at lunchtime and the ounces of water taken from water sources.

### Outcome/predictor variables and covariates

The primary outcome was students’ lunchtime water consumption. Beverage intake surveys ascertained whether students drank any water at lunchtime (ie, water fountain, tap water from home, nonflavored bottled water, and water from the new water source [for follow-up only]). If students responded yes, they were then asked to indicate 1) the brand of the water, if applicable; 2) the location where they obtained the water (vending machine, free with lunch, from a friend at school, brought it from home, bought it on the way to school, someplace else); and 3) how much water they drank (a few sips, less than a glass or less than half a bottle, 1 glass or one-half a bottle, 2 glasses or 1 bottle, more than 2 glasses or more than 1 bottle). Because the data were skewed (ie, a large proportion of students reported drinking no or only a few sips of beverages), survey responses were dichotomized into 1) drank more than a few sips or 2) drank a few sips or less.

Researchers estimated the proportion of students drinking free water at lunch by dividing the number of students observed using free water sources by the total number of students in attendance. To obtain the ounces of free water consumed per student at lunchtime, researchers measured the volume of water taken from dispensers (manual measurements) and bottleless coolers (totalizing water flow meters) at lunchtime and divided that number by the students in daily attendance. Researchers did not obtain measurements of water taken from fountains in control schools.

Secondary outcomes included students’ lunchtime consumption of SSBs, 100% fruit juice, and milk. Students reported whether they drank certain beverages at lunchtime and, if so, provided the beverage’s brand, where they obtained the beverage, and the quantity of beverage they consumed. SSB consumption included flavored water with sweeteners, flavored milk, nondiet sodas, nondiet sports drinks, energy drinks, and other sugary or sweetened drinks. The main predictor was school randomization to receive 1) dispensers and promotion, 2) a cooler and promotion, or 3) the control.

Covariates were student sociodemographic characteristics associated with water intake in previous studies, including age (22), sex (23), race/ethnicity (African American, Latino/Hispanic, Asian/Pacific Islander, other) (16), language spoken at home (English only, English and another language, no English) (24), and being US-born versus being foreign-born (25).

### Cost estimates for intervention components

Researchers used methods from previous studies to estimate the cost of providing and promoting water in intervention schools (26). For comparability, estimates were calculated for a 24-hour period for schools with 500 students and 180 operational school days per year. Researchers used the average amount of water consumed during observations to estimate water intake. They used purchase prices to determine water source costs and estimated cup costs using the single cup cost multiplied by the number of cups used during the intervention. Researchers estimated labor time through logs of school staff time during week 6 of the intervention in which they recorded the number of daily minutes they spent on tasks related to serving the water. To estimate the cost of this labor, researchers multiplied the average daily hours spent providing water by the average hourly wage for school staff in custodial and cafeteria positions in the relevant California counties.

### Data analysis

Data were analyzed from February 2013 to August 2015. Means, standard deviations (SDs), and proportions were calculated for school and student sociodemographics. Analysis of variance and χ^2^ tests were conducted to examine differences by study arm in continuous and categorical sociodemographic characteristics, respectively. Mixed-effects linear regression was used to estimate differences in the change from baseline to postintervention in 1) the students who took free water from intervention water sources and 2) the volume of water taken from water sources at lunchtime between intervention and control schools. Mixed-effects logistic regression models examined differences in the change from baseline to postintervention in the proportion of students who reported that they drank more than a few sips of various beverages during lunchtime in intervention and control schools. Models included primary dependent variables, time period indicators, intervention group, the interaction of time period and intervention group, and student sociodemographic characteristics (age, sex, race/ethnicity, language spoken at home, and US-born status). Random effects for student and school accounted for clustering of students in schools. Missing data comprised less than 4% in all cases. Stata/MP version 13 (StataCorp LP) was used for analyses.

## Results

**Figure 1:**
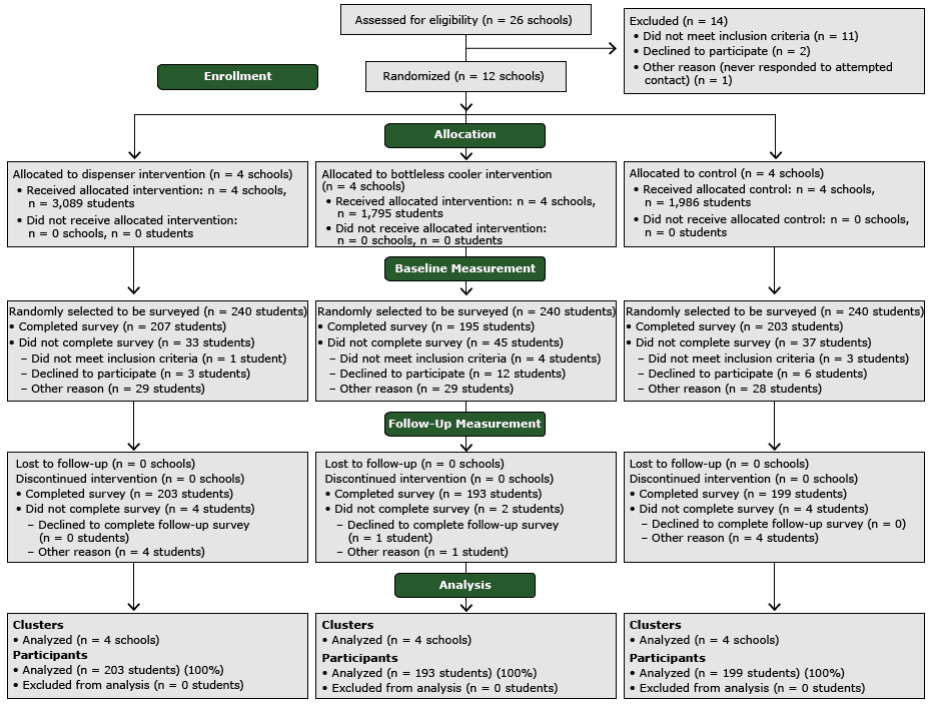
Consolidated standards of reporting trial diagram of study participants from middle schools, San Francisco Bay Area, 2013.

Twenty-six schools were approached for the study. Two declined because of concerns that the study evaluation would be disruptive to instructional time, 11 already had a nonfountain water source in their cafeteria, and 1 never responded (Figure 1). Of 720 students sampled to complete beverage intake surveys, 605 (84%) took baseline surveys. Reasons why students did not take surveys included absence (45%), student refusal (18%), not showing up to the survey location (22%), ineligibility because of low literacy (6%), relocation out of the school (7%), and parent refusal (2%). Of students who took baseline surveys, 595 (98%) took follow-up surveys. Despite random assignment, there were fewer African American students in schools with dispensers (*P* = .002) than in schools with coolers or schools that were controls and more male students in schools with dispensers (*P* = .02) (Table 1) than in schools with coolers or schools that were controls.

**Table 1:** Characteristics of Middle School Students, by Study Arm, Efficacy and Cost of Water Delivery Systems, San Francisco Bay Area, 2013^a^

Characteristic	Control/Fountain	Dispenser	Cooler
**School level^b^**	**n = 1,978**	**n = 3,054**	**n = 1,412**
Race/ethnicity			
Latino/Hispanic	835 (42.2)	1,866 (61.1)	621 (44.0)
African American	579 (29.3)	454 (14.9)	416 (29.5)
Asian/Pacific Islander	314 (15.9)	377 (12.3)	188 (13.3)
Other	250 (12.6)	357 (11.7)	187 (13.2)
English learners	504 (25.5)	796 (26.1)	362 (25.6)
Free and reduced-price meal eligibility	1,423 (71.9)	2,374 (77.7)	1,006 (71.2)
**Student level**	**n = 199**	**n = 203**	**n = 193**
Age, y, mean (SD)	12.7 (1.0)	12.6 (0.9)	12.6 (0.9)
Male sex^c^	83 (41.7)	113 (55.7)	85 (44.0)
Race/ethnicity^d^^a^			
Latino/Hispanic	108 (54.3)	126 (62.1)	101 (52.3)
African American^c^	43 (21.6)	25 (12.3)	51 (26.4)
Asian/Pacific Islander	36 (18.1)	30 (14.8)	30 (15.5)
Other	36 (18.1)	32 (15.8)	38 (19.7)
Languages spoken at home			
English only	102 (51.3)	95 (46.8)	105 (54.4)
English plus another language	34 (17.1)	32 (15.8)	28 (14.5)
No English	63 (31.7)	76 (37.4)	60 (31.1)
US-born	157 (78.9)	164 (80.8)	150 (77.7)

Abbreviation: SD, standard deviation.

a Analysis of variance and χ^2^ tests conducted to examine differences among continuous and categorical socioeconomic characteristics across study arms. Values presented as no. (%), unless otherwise indicated.

b Data obtained from Education Data Partnership (www.ed-data.org).

c Significant findings (*P* *≤* .05).

d Values for race/ethnicity exceed the total values for n, because students could choose more than one answer.

**Figure 2:**
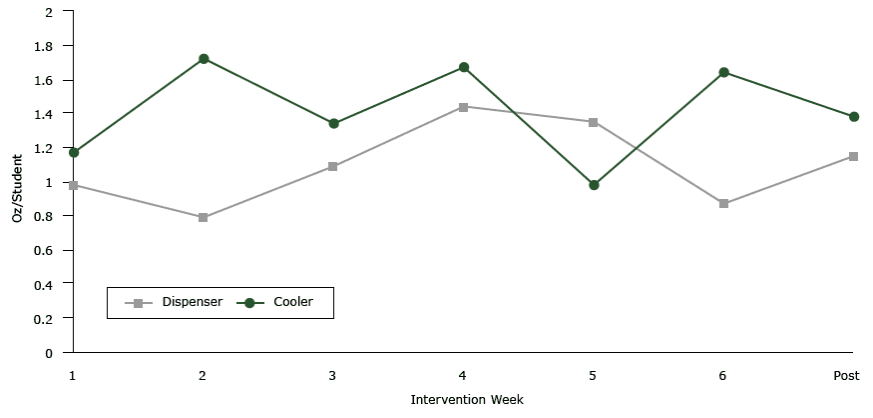
Measurements of water taken from free water sources in cafeterias at lunch in San Francisco Bay Area Middle Schools, 2013. The mean ounces of water per student taken from intervention water sources at lunch during the intervention period was not significantly different in schools with water dispensers than in schools with bottleless water coolers (*P *= .19). Water taken from traditional fountains in control schools was not measured. **Table:** 

During observations over the study period, the mean (SD) ounces per student of water taken from cafeteria water sources at lunchtime was not significantly higher in schools with dispensers than in schools with bottleless coolers (1.0 [0.6] vs 1.5 [1.6]; *P* = .19) (Figure 2). Among students drinking water, the mean (SD) ounces of water consumed per student postintervention did not differ in schools with dispensers versus in schools with coolers (7.3 [1.4] vs 6.9 [1.7]; *P* = .73). There was a trend for an increase in the mean percentage of students observed accessing cafeteria water sources at lunchtime in schools with appealing water sources, cups, and promotion compared with control schools with traditional drinking fountains (dispenser vs control, 11.9%; 95% CI, −0.6 to 0.3; *P* = .19; cooler vs control, 17.3%; 95% CI, −0.01 to 0.4; *P* = .06).

Intervention Week	Dispenser	Cooler
1	0.98	1.17
2	0.79	1.72
3	1.09	1.34
4	1.44	1.67
5	1.35	0.98
6	0.87	1.64
Post	1.15	1.38

During the study period, there was an increase in number of students who drank more than a few sips of water at lunchtime in schools with dispensers and coolers compared with controls (Table 2). After adjustment for covariates, this change remained significant in schools with dispensers (adjusted odds ratio [AOR] = 3.1; 95% CI, 1.4–6.7; *P* = .004) but not in schools with coolers (AOR = 1.7; 95% CI, 0.8–3.7; *P* = .17). There were no significant differences in the change in water consumption reported by students from preintervention to postintervention between schools with dispensers versus coolers (AOR = 1.7; 95% CI, 0.8–3.5; *P* = .14). The intervention did not lead to significant changes in intake of milk, 100% fruit juice, or SSBs. Sensitivity analyses excluding schools that received hydration stations from a source outside of the study did not significantly alter results.

**Table 2:** Intervention Effect on the Percentage of Students (n = 595) Reporting Intake of Various Beverages at Lunch, From Baseline to Follow-Up, Efficacy and Cost of Water Delivery Systems, San Francisco Bay Area Middle Schools, 2013^a^

Variable	Baseline Unadjusted Mean % (SD)	Follow-up Unadjusted Mean % (SD)	Crude Difference	Difference in Difference	AOR (95% CI)^b^	*P* Value
**Students drinking water**						
Dispenser	31.7 (4.7)	49.9 (5.0)	18.2	18.9	3.1 (1.4–6.7)	.004
Cooler	39.2 (4.9)	49.0 (5.0)	9.8	10.7	1.7 (0.8–3.7)	.17
Any intervention^c^	35.3 (4.8)	49.4 (5.0)	14.1	14.9	2.3 (1.2–4.5)	.02
Control	34.8 (4.8)	34.0 (4.7)	−0.8	NA	1 [Reference]	NA
**Students drinking milk**						
Dispenser	16.9 (3.8)	13.8 (3.5)	−3.1	−5.1	0.6 (0.2–1.4)	.23
Cooler	21.0 (4.1)	22.3 (4.2)	1.3	−0.7	0.9 (0.4–2.2)	.86
Any intervention	18.9 (3.9)	18.0 (3.8)	−0.9	−2.9	0.7 (0.4–1.5)	.43
Control	31.0 (4.6)	33.0 (4.7)	2.0	NA	1 [Reference]	NA
**Students drinking juice**						
Dispenser	12.7 (3.3)	12.8 (3.4)	0.1	1.4	1.2 (0.3–4.4)	.78
Cooler	11.3 (3.2)	13.3 (3.4)	2.0	3.3	2.4 (0.6–9.1)	.19
Any intervention	12.1 (3.3)	13.1 (3.4)	1.0	2.3	1.7 (0.5–5.2)	.37
Control	13.4 (3.4)	12.1 (3.3)	−1.3	NA	1 [Reference]	NA
**Students drinking sugar-sweetened beverages**						
Dispenser	28.0 (4.5)	24.1 (4.3)	−3.9	0.6	1.2 (0.6–2.4)	.71
Cooler	30.8 (4.6)	27.2 (4.5)	−3.6	1.0	1.1 (0.5–2.3)	.78
Any intervention	29.4 (4.6)	25.7 (4.4)	−3.7	0.8	1.1 (0.6–2.1)	.70
Control	35.5 (4.8)	31.0 (4.6)	−4.5	NA	1 [Reference]	NA

Abbreviations: AOR, adjusted odds ratio; CI, confidence interval; NA, no applicable data; SD, standard deviation.

a Students (n = 595) classified as drinking beverages are those who reported drinking more than a few sips of beverages.

b Mixed-effects logistic regression models included reported consumption of more than a few sips of beverages, intervention status, time period (preintervention vs postintervention), interaction of intervention status and time, age, sex, race/ethnicity, US-born status, and language spoken at home. Significance set at *P* ≤ .05.

c “Any intervention” refers to schools that had either water dispensers or bottleless water coolers.

Although the upfront annual cost in year 1 of installing and promoting water dispensers was lower than that of providing and promoting bottleless water coolers, the average cost over a 5-year period was similar for both interventions at approximately $0.04 per student per day (Table 3).

**Table 3:** Cost Estimates of Serving and Promoting Water at Lunch in San Francisco Bay Area Middle Schools, 2013^a^^,^^b^

Cost Category	Dispenser	Cooler
**Water**		
Gallon capacity	3	Unlimited (1 gallon for chilled water)
Water, oz/student at lunch	1.0	1.5
Cost per year^c^	3.7	5.4
**Infrastructure**		
Total source cost	204.6	345.0
Unit(s)	141.4 (for 2 units)	345.0
Installation	NA	Included in unit price
Food cart for transporting dispenser	63.2	NA
Infrastructure cost per 5 years	628.6	345.0
**Other**		
Cups/y	678.2	1,309.0
Electricity/y^d^	435.5	1,562.1
Labor/y	2,054.2	595.7
Promotion/y^e^	155.4	155.4
Water testing and remediation^f^/5 years	47.6	47.6
**Total**		
Year 1	3,579.1	4,020.2
Annual average, years 2–5	3,480.5	3,634.1
Annual average, years 1–5	3,500.2	3,711.3
Cost per student per day	0.04	0.04
Cost per ounce of water consumed	0.04	0.03

Abbreviation: NA, not applicable.

a All costs are reported in US dollars.

b Based on 500 students and 180 days of school.

c Based on average price per gallon charged to study schools by their local water supplier.

d Based on units using 2.51kW/h from Culligan manual and average price per kWh charged to study schools by their local electric utility.

e Estimates for promotion include cost of a large wall decal, 6 posters, and prizes (eg, 100 stickers, and promotional pins).

f Actual average cost across study schools to test for and remediate lead in water.

## Discussion

This is the first randomized controlled trial to compare the efficacy and cost of different water delivery sources in increasing students’ intake of water in school cafeterias. Providing dispensers with cups in school cafeterias and promoting their use led to nearly a 20% increase in the proportion of students who drank water at lunch as compared with school cafeterias with only traditional drinking fountains. Previous water access and promotion interventions in US schools led to more modest increases in the proportion of students drinking water (approximately 10%). In one of those studies, cups were not provided next to the cafeteria water source (6). In a second study, no promotion was provided (7). In a third study, signage and disposable cups were provided next to traditional drinking fountains (15). The greater increase observed in this study may be a result of the combined effect of safe and appealing water sources, provision of cups, and promotion of water intake.

Most US schools meet the requirement for water in the school food service area through traditional drinking fountains (12). Although school administrators can provide more appealing water sources, they may worry about the cost of such improvements. This study suggests that providing water in small dispensers with cups may be a feasible, low-cost option for schools until they secure funds for a more sustainable water bottle filling station. In this study, schools were able to absorb nearly 70% of the cost of providing the dispensers (including both energy and labor costs) by using preexisting refrigerators (on hand to store perishable school meal items) to chill water dispensers and by having existing food service staff fill and clean dispensers.

Schools may worry that offering free drinking water will decrease the sales of bottled water from school vending machines, which are present in 54% of middle schools and 90% of US high schools (27). Although our study did not examine how free water access affected vending machine sales of bottled water, schools should also consider costs associated with operating vending machines (annual average energy cost of $313 per machine and a reduction in revenue from school meal programs) (28).

Schools may also be concerned about a water program’s potential to decrease students’ intake of milk. In this study, the intervention did not result in a significant decrease in the proportion of students drinking milk at lunch. A study in New York City that compared 10 public schools that received water dispensers to 9 control schools demonstrated a decline in students who took milk at lunch in intervention schools at 3 months (−6.7 per 100 events), but that change was not sustained at the 1-year follow-up (29). Studies with a longer follow-up period should explore the effect of school-based water programs on milk intake throughout the day.

One critical goal of providing access to water in schools is to provide a substitute for SSBs. Because students drank only 1 to 1.5 ounces of water at lunchtime, it is not surprising that this study, like others, had no significant effect on students’ SSB intake (5,6). In a previous study in which cups and signage were placed near drinking fountains, water consumption at lunchtime was slightly lower at 0.7 ounces per student (15). The reach of this study’s interventions in improving students’ water intake may be limited because many students do not eat lunch in the cafeteria where water sources are located. Moreover, despite bans on SSB sales on school campuses, students in this study still drank SSBs that they brought from home or purchased on the way to school (30). To have a substantial effect on students’ SSB intake, more comprehensive interventions that target access to SSBs that students can bring in from home or from vendors near schools are needed.

There were several limitations of this study. Many limitations relate to study design and methods, including a small sample size (4 schools in each study category), limited generalizability to dissimilar schools, a short intervention period, 1-day rather than 2-day observations of students’ water intake, lack of long-term follow-up, and the absence of data on health outcomes. Social desirability bias could have led to students overreporting water intake on self-reported surveys; however, in observations, we documented similar trends in the percentage of students drinking water, increasing our confidence in this study’s results.

Despite these limitations, results from the multiple evaluation methods used in this study (students’ self-reported beverage intake and observational data) support the positive effect of these low-cost water promotion interventions in increasing students’ water intake. Future studies should also explore the effectiveness of such interventions in improving health and learning outcomes among children.

Offering more appealing water sources and providing cups in school cafeterias can increase students’ intake of water at lunchtime. To encourage students to substitute their intake of SSBs with water, however, it may be important not only to rethink the traditional drinking fountain but also to strengthen language about water and SSBs in school wellness policies, to offer appealing drinking water throughout the school campus, and to promote intake of water instead of SSBs.

## References

[1] Krassas GE , Tzotzas T . Do obese children become obese adults: childhood predictors of adult disease. Pediatr Endocrinol Rev 2004;1(Suppl 3):455–9. 16444174

[2] Malik VS , Popkin BM , Bray GA , Després JP , Hu FB . Sugar-sweetened beverages, obesity, type 2 diabetes mellitus, and cardiovascular disease risk. Circulation 2010;121(11):1356–64. 10.1161/CIRCULATIONAHA.109.876185 20308626 PMC2862465

[3] Marshall TA , Levy SM , Broffitt B , Warren JJ , Eichenberger-Gilmore JM , Burns TL , Dental caries and beverage consumption in young children. Pediatrics 2003;112(3 Pt 1):e184–91. 10.1542/peds.112.3.e184 12949310

[4] Chriqui JF , Pickel M , Story M . Influence of school competitive food and beverage policies on obesity, consumption, and availability: a systematic review. JAMA Pediatr 2014;168(3):279–86. 10.1001/jamapediatrics.2013.4457 24473632

[5] Muckelbauer R , Libuda L , Clausen K , Toschke AM , Reinehr T , Kersting M . Promotion and provision of drinking water in schools for overweight prevention: randomized, controlled cluster trial. Pediatrics 2009;123(4):e661–7. 10.1542/peds.2008-2186 19336356

[6] Patel AI , Bogart LM , Elliott MN , Lamb S , Uyeda KE , Hawes-Dawson J , Increasing the availability and consumption of drinking water in middle schools: a pilot study. Prev Chronic Dis 2011;8(3):A60. 21477500 PMC3103565

[7] Schwartz AE , Leardo M , Aneja S , Elbel B . Effect of a school-based water intervention on child body mass index and obesity. JAMA Pediatr 2016;170(3):220–6. 10.1001/jamapediatrics.2015.3778 26784336 PMC4977575

[8] Institute of Medicine.. Accelerating progress in obesity prevention: solving the weight of the nation. Washington (DC): The National Academies Press; 2012.

[9] Centers for Disease Control and Prevention. School health guidelines to promote healthy eating and physical activity. http://www.cdc.gov/healthyschools/npao/pdf/mmwr-school-health-guidelines.pdf. Accessed April 14, 2016.

[10] Kenney EL , Long MW , Cradock AL , Gortmaker SL . Prevalence of inadequate hydration among US children and a disparities by gender and race/ethnicity: National Health and Nutrition Examination Survey, 2009–2012. Am J Public Health 2015;105(8):e113–8. 10.2105/AJPH.2015.302572 26066941 PMC4504329

[11] Healthy, Hunger-Free Kids Act of 2010, 42 USC 1751, §203 (2010).

[12] Hood NE , Turner L , Colabianchi N , Chaloupka FJ , Johnston LD . Availability of drinking water in US public school cafeterias. J Acad Nutr Diet 2014;114(9):1389–95. 10.1016/j.jand.2014.02.001 24726348

[13] Onufrak SJ , Park S , Sharkey JR , Merlo C , Dean WR , Sherry B . Perceptions of tap water and school water fountains and association with intake of plain water and sugar-sweetened beverages. J Sch Health 2014;84(3):195–204. 10.1111/josh.12138 24443781 PMC4559844

[14] Patel AI , Bogart LM , Klein DJ , Burt Cowgill , Uyeda KE , Hawes-Dawson J , Middle school student attitudes about school drinking fountains and water intake. Acad Pediatr 2014;14(5):471–7. 10.1016/j.acap.2014.05.010 25169158 PMC4193898

[15] Kenney EL , Gortmaker SL , Carter JE , Howe MC , Reiner JF , Cradock AL . Grab a cup, fill it up! An intervention to promote the convenience of drinking water and increase student water consumption during school lunch. Am J Public Health 2015;105(9):1777–83. 10.2105/AJPH.2015.302645 26180950 PMC4539814

[16] Beck AL , Patel A , Madsen K . Trends in sugar-sweetened beverage and 100% fruit juice consumption among California children. Acad Pediatr 2013;13(4):364–70. 10.1016/j.acap.2013.02.010 23688439 PMC3706491

[17] National Center for Health Statistics. Obesity and socioeconomic status in children and adolescents: United States, 2005–2008. NCHS Data Brief. Number 51. http://files.eric.ed.gov/fulltext/ED530165.pdf. Accessed April 4, 2016. 21211166

[18] Brennan LK , Brownson RC , Orleans CT . Childhood obesity policy research and practice: evidence for policy and environmental strategies. Am J Prev Med 2014;46(1):e1–16. 24355679 10.1016/j.amepre.2013.08.022 PMC4762255

[19] Environmental Protection Agency. 3T’s for reducing lead in drinking water in schools. http://www.epa.gov/ogwdw/schools/pdfs/lead/toolkit_leadschools_guide_3ts_leadschools.pdf. Accessed July 31, 2014.

[20] Paxton A , Baxter SD , Fleming P , Ammerman A . Validation of the school lunch recall questionnaire to capture school lunch intake of third- to fifth-grade students. J Am Diet Assoc 2011;111(3):419–24. 10.1016/j.jada.2010.11.017 21338742

[21] Patel AI , Chandran K , Hampton KE , Hecht K , Grumbach JM , Kimura AT , Observations of drinking water access in school food service areas before implementation of federal and state school water policy, California, 2011. Prev Chronic Dis 2012;9:E121. 22765930 10.5888/pcd9.110315 PMC3468310

[22] Han E , Powell LM . Consumption patterns of sugar-sweetened beverages in the United States. J Acad Nutr Diet 2013;113(1):43–53. 10.1016/j.jand.2012.09.016 23260723 PMC3662243

[23] Ogden CL , Kit BK , Carroll MD , Park S . Consumption of sugar drinks in the United States, 2005-2008. NCHS Data Brief 2011;(71):1–8. 22617020

[24] Ayala GX , Baquero B , Klinger S . A systematic review of the relationship between acculturation and diet among Latinos in the United States: implications for future research. J Am Diet Assoc 2008;108(8):1330–44. 10.1016/j.jada.2008.05.009 18656573 PMC3727241

[25] Sharkey JR , Johnson CM , Dean WR . Nativity is associated with sugar-sweetened beverage and fast-food meal consumption among Mexican-origin women in Texas border colonias. Nutr J 2011;10(1):101. 10.1186/1475-2891-10-101 21962014 PMC3196692

[26] Cradock AL , Wilking CL , Olliges SA , Gortmaker SL . Getting back on tap: the policy context and cost of ensuring access to low-cost drinking water in Massachusetts schools. Am J Prev Med 2012;43(3, Suppl 2):S95–101. 10.1016/j.amepre.2012.05.016 22898169

[27] Bridging the Gap. School policies and practices to improve health and prevent obesity: National Secondary School Survey results. http://www.bridgingthegapresearch.org/_asset/mrvfbr/SS_2015_report.pdf. Accessed April 14, 2016.

[28] Public Health Advocacy Institute. The hidden cost of school beverages vending machines. http://www.phaionline.org/wp-content/uploads/2012/06/HiddenEnergyCostofVending.pdf. Accessed April 2, 2015.

[29] Elbel B , Mijanovich T , Abrams C , Cantor J , Dunn L , Nonas C , A water availability intervention in New York City public schools: influence on youths’ water and milk behaviors. Am J Public Health 2015;105(2):365–72. 10.2105/AJPH.2014.302221 25521867 PMC4318331

[30] Grummon AH , Oliva A , Hampton KE , Patel AI . Association between student purchases of beverages during the school commute and in-school consumption of sugar-sweetened beverages, San Francisco Bay Area, 2013. Prev Chronic Dis 2015;12:E220. 26679489 10.5888/pcd12.150306 PMC5241631

